# Circulating microRNAs: Potential Markers of Cardiotoxicity in Children and Young Adults Treated With Anthracycline Chemotherapy

**DOI:** 10.1161/JAHA.116.004653

**Published:** 2017-04-04

**Authors:** Kasey J. Leger, David Leonard, Danelle Nielson, James A. de Lemos, Pradeep P.A. Mammen, Naomi J. Winick

**Affiliations:** ^1^ Department of Pediatrics Seattle Children's Hospital University of Washington School of Medicine Seattle WA; ^2^ Department of Clinical Research Children's Medical Center Dallas TX; ^3^ Department of Pediatrics University of Texas Southwestern Medical Center Dallas TX; ^4^ Department of Internal Medicine University of Texas Southwestern Medical Center Dallas TX; ^5^ Heart Failure, Ventricular Assist Device & Heart Transplant Program University of Texas Southwestern Medical Center Dallas TX

**Keywords:** anthracycline, cancer, cardiotoxicity, microRNA, pediatric, Cardiomyopathy, Biomarkers, Pediatrics, Complications

## Abstract

**Background:**

Biomarkers for early detection of anthracycline (AC)‐induced cardiotoxicity may allow cardioprotective intervention before irreversible damage. Circulating microRNAs (miRNAs) are promising biomarkers of cardiovascular disease, however, have not been studied in the setting of AC‐induced cardiotoxicity. This study aimed to identify AC‐induced alterations in plasma miRNA expression in children and correlate expression with markers of cardiac injury.

**Methods and Results:**

Candidate plasma profiling of 24 miRNAs was performed in 33 children before and after a cycle of AC (n=24) or noncardiotoxic chemotherapy (n=9). Relative miRNA changes between the pre‐ and postcycle time points (6, 12, and 24 hours) were determined within each treatment group and compared across groups. Plasma miRNA expression patterns were further explored with respect to AC dose and high‐sensitivity troponin T. Greater chemotherapy‐induced dysregulation was observed in this panel of candidate, cardiac‐related plasma miRNAs in patients receiving anthracyclines compared with those receiving noncardiotoxic chemotherapy (24‐hour MANOVA;* P*=0.024). Specifically, plasma miRs‐29b and ‐499 were upregulated 6 to 24 hours post‐AC, and their postchemotherapy expression significantly correlated with AC dose. Patients with acute cardiomyocyte injury (high‐sensitivity troponin T increase ≥5 ng/L from baseline) demonstrated higher expression of miR‐29b and miR‐499 post‐AC compared with those without.

**Conclusions:**

In this pilot study, cardiac‐related plasma miRNAs are dysregulated following ACs. Plasma miR‐29b and ‐499 are acutely elevated post‐AC, with dose response relationships observed with anthracycline dose and markers of cardiac injury. Further evaluation of miRNAs may provide mechanistic insight into AC‐induced cardiotoxicity and yield biomarkers to facilitate earlier intervention to mitigate cardiotoxicity.

## Introduction

Anthracycline (AC)‐related cardiotoxicity is among the most significant long‐term threats to cancer survivors, with cardiac events comprising the most common nonmalignant cause of death in this population.^1^, ^2^ The estimated prevalence of cardiomyopathy in 50‐year‐old survivors of childhood cancer exposed to cardiotoxic chemotherapy or radiation is 21%.^3^ Once AC‐induced cardiomyopathy develops, it is commonly progressive, with no definitive treatment other than implantation of a left ventricular assist device and/or heart transplantation.^4^, ^5^, ^6^ Currently, methods to detect subclinical cardiac injury early in the course of progressive cardiotoxicity are lacking. Early identification of patients at high risk for cardiac morbidity could allow implementation of cardioprotective measures during a stage when these therapies are likely to impact cardiac outcome.^7^, ^8^ Pediatric studies exploring anthracycline (AC)‐induced trends in cardiac troponin demonstrate a correlation between early troponin elevations and late echocardiographic abnormalities.^9^, ^10^ However, studies have yet to reveal quantitative trends in troponin levels that specifically predict late cardiac outcomes, and thus troponins are insufficient to guide cardioprotective intervention.

MicroRNAs (miRNAs) are short, noncoding RNAs that regulate fundamental cellular processes and play a critical role in cardiovascular development and disease.^11^, ^12^ Circulating miRNAs are promising biomarkers of cardiovascular disease attributed to their surprisingly high stability in plasma, tissue‐ or cell‐specific distribution, and ease of quantitative measurement.^13^, ^14^ The demonstrated sensitivity and specificity of miRNAs in various cardiovascular disease states indicate a potential role for miRNAs in early detection of cardiotoxicity.^15^

Although the role of miRNAs in modulating anthracycline‐induced cardiac injury has been demonstrated in animal models,^16^, ^17^, ^18^ little is known about AC‐induced alterations in plasma miRNAs. The aim of this study was to explore acute AC‐induced alterations in plasma miRNA expression in children and correlate miRNA expression with known markers or predictors of cardiac injury. We hypothesized that children receiving AC chemotherapy would demonstrate a unique plasma miRNA profile compared with controls receiving noncardiotoxic chemotherapy, and that this plasma miRNA signature could identify children with AC‐induced cardiac injury.

## Methods

### Study Design

**Figure 1:**
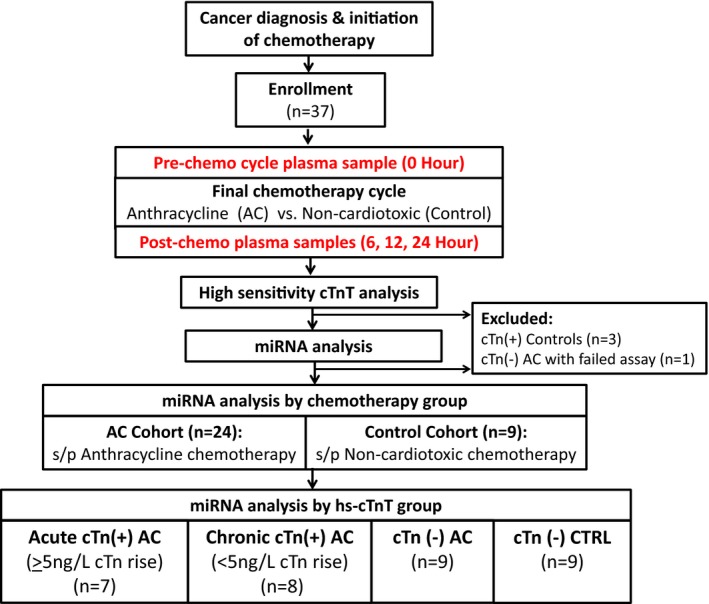
Study schematic and patient classification. Children being treated with chemotherapy for cancer were enrolled before a cycle of anthracycline (AC) or noncardiotoxic (Control) chemotherapy. Plasma was collected before and at several points following the chemotherapy cycle. Plasma miRNA expression was evaluated according to chemotherapy group (AC vs noncardiotoxic) and troponin elevation. AC indicates anthracycline; Acute cTn(+), patients with hs‐cTnT ≥14 ng/L whose post‐AC cTnT was ≥5 ng/L above the precycle‐AC baseline; Chronic cTnT(+), patients with hs‐cTnT ≥14 ng/L whose pre‐ and postcycle‐AC troponin levels remained stable (<5 ng/L rise from baseline); cTn(−), patients with plasma cTnT level <14 ng/L at all time points; cTn(+), patients with plasma hs‐cTnT level ≥14 ng/L at any time point (0, 6, 12, or 24 hours); CTRL, control group; hs‐cTnT, high‐sensitivity cardiac troponin T assay.

This was a prospective cohort study of patients aged <18 years receiving AC or noncardiotoxic chemotherapy (controls) at Children's Medical Center Dallas (Figure 1), approved by the University of Texas Southwestern Institutional Review Board (Dallas, TX). Patients with a history of congenital heart disease, Down syndrome, or those who received radiation to the heart were ineligible. Patients receiving AC, high‐dose cyclophosphamide (>2000 mg/m^2^ per cycle or >1200 mg/m^2^ per dose), or tyrosine kinase inhibitors or those with any evidence of cardiac injury (high‐sensitivity cardiac troponin T [hs‐cTnT] >14 ng/L) were not eligible for the control group. Potential subjects were identified and referred for recruitment by their primary oncology provider. Those meeting eligibility criteria were approached for enrollment and informed consent was obtained. In all cases, AC chemotherapy was given by bolus infusion over 15 to 30 minutes. In most (n=21 of 25) cases, the AC group underwent sample collection during their final cycle of AC; controls underwent collection during a late treatment cycle of a noncardiotoxic chemotherapy regimen. In the AC group, a “pre‐AC” cycle baseline sample was drawn before the first cycle dose of AC, with post‐AC sample collection 6, 12, and 24 hours following the final AC dose. The 12‐hour sample was only drawn from patients who were hospitalized (collected in 24 of 33 patients). Because this study was designed to assess miRNA alteration following the maximal cumulative AC exposure, all but 1 patient in the AC group had anthracycline exposure before to their “day 0” precycle baseline. Controls underwent the same schedule of plasma collection timed around a dose of noncardiotoxic chemotherapy.

### Sample Processing, RNA Isolation, and MicroRNA Profiling

Plasma samples were collected either by peripheral venipuncture or from a central line (see Data S1 for details). Briefly, RNA was isolated using a miRNeasy serum/plasma microRNA isolation kit (Qiagen, Hilden, Germany) and reverse transcribed using the Taqman miRNA reverse transcription kit. Custom TaqMan Array MicroRNA Cards (Product# 4346799, Format 32; Applied Biosystems, Foster City, CA) were designed to assay 24 candidate miRNAs and 7 miRNAs for quality assessment and normalization. Candidate miRNAs were selected based on their demonstrated roles as biomarkers or mediators of cardiovascular disease (Table S1). The final endogenous miRNA controls used for miRNA normalization were chosen based on stability score (Table S2).

### High‐Sensitivity Troponin Assay

hs‐cTnT was measured using an automated immunoassay (Troponin T hs STAT, Elecsys‐2010; Roche Diagnostics, Indianapolis, IN).^19^ Five nanograms per liter represents the limit of detection for this assay. An absolute threshold of ≥14 ng/L is considered abnormal for this assay.^20^, ^21^ Assessment of change in troponin over time is recommended to discriminate patients with and without acute cardiomyocyte injury.^22^, ^23^ Therefore, we considered patients to have a significant chemotherapy‐induced troponin rise if hs‐cTnT 6 to 24 hours after chemotherapy was at least 5 ng/L greater than the precycle baseline. Patients were categorized into 3 hs‐cTnT categories, those with: acute hs‐cTnT elevations (hs‐cTnT ≥14 ng/L at any time point, plus ≥5 ng/L rise from baseline); chronic elevation (hs‐cTnT >14 ng/L at any time point, but <5 ng/L rise from baseline); or normal (<14 ng/L at all time points).

### Statistical Analysis

Comparisons across groups were made using Fisher's exact tests for nominal characteristics and rank‐sum tests for continuous characteristics. Individual patient troponin values across 0‐, 6‐, 12‐, and 24‐hour time points were plotted by type of chemotherapy received (Figure S1). Differences in miRNA regulation from baseline were compared between AC and control groups at each time point (6, 12, and 24 hours) using linear mixed‐effects regression models for each of the 24 target miRNAs. The models included fixed effects of group (anthracycline versus noncardiotoxic chemotherapy or cTnT rise ≥5 ng/L versus <5 ng/L), time (0, 6, 12, and 24 hours), condition (target miRNA versus control miRs‐140 and ‐484), and all 2‐ and 3‐factor cross‐terms. The models included random intercept, time, condition, and time×condition by patient effects to account for correlated cycle threshold (Ct) levels, changes over time, differences between conditions, and differences between changes over time by condition within patients, respectively. Additionally, MANOVA was applied to miRNA levels averaged by patient over the three Ct replicates at the 6‐ and 24‐hour time points and the 2 control conditions to test the hypothesis of no overall group effect on the entire panel of candidate miRNAs. The number of samples collected at 12 hours was insufficient to perform this test.

Twenty‐four miRNAs were measured in the cohort and 3 were selected for further analyses based on their consistent differential regulation between treatment groups with between group *P*<0.05 (miRs‐1 and ‐29b) or between group *P*<0.1 with miRNA with fold‐change >2 at all time points in the AC group with no change in the treatment group (miR‐499). These miRNAs were subsequently compared across troponin groups (hs‐cTnT rise ≥5 ng/L versus <5 ng/L) using similar regression modeling to that used to analyze treatment groups. Two miRNAs with differential expression between troponin groups were further analyzed to explore their relationship with relevant covariates including interim and cumulative AC dose, wherein normalized miRNA cycle threshold values at each time point were regressed linearly and separately on each covariate using data averaged over the 3 Ct replicates by patient and time. Finally, logistic regression models were fit in the subgroup of patients receiving ACs to explore the sensitivity and specificity of normalized miR‐29b and ‐499 expression for predicting patients with a significant rise in hs‐cTnT post‐AC (≥5 ng/L above baseline). These models used average Ct data by patient, time, and miRNA to construct receiver operator characteristic curves.

A priori, we targeted recruitment of 34 patients (24 AC‐exposed; 10 controls), which would provide 80% power to detect a 2‐fold mean group change in miRNA expression from baseline between groups, with an alpha of 0.05. Reported *P* values were not adjusted for multiple comparisons. All analyses were programmed in SAS/STAT software (version 9.4; SAS Institute Inc., Cary, NC). Additional details on both mRNA and troponin assays as well as the statistical modeling can be found in Data S1.

## Results

### Clinical Data and High‐Sensitivity Troponin T Assay

A total of 37 patients were enrolled (n=25 AC and n=12 control), 33 of whom were included in the miRNA analysis (n=24 AC and n=9 control). Three patients receiving intermediate‐dose cyclophosphamide (1200 mg/m^2^) were excluded from the control group because of low‐level elevations in hs‐cTnT, in order to maintain a control group free of any evidence of cardiac injury. Additionally, 1 patient receiving doxorubicin had uniformly low Ct values (global mean <2 SD below that of the entire group) in 3 of the 4 sample time points, thus was excluded because of technical problems with the sample/assay. The AC group contained patients with various diagnoses (Table) who received doxorubicin (n=15), mitoxantrone (n=7), daunorubicin (n=1), or idarubicin (n=1) fractionated over 1 to 5 days. The mean cumulative anthracycline dose before baseline plasma collection and the individual cycle dose around which the plasma samples were collected were 321±30 and 102±13 mg/m^2^ in doxorubicin equivalents, respectively. Eight patients received dexrazoxane in addition to their cycle dose of anthracycline, either per institutional protocol (n=7) or for left ventricular dysfunction following a previous anthracycline cycle (n=1). The control group contained patients with a variety of tumor types who received non‐AC containing chemotherapeutic regimens, including cisplatin, carboplatin, cyclophosphamide, ifosfamide, etoposide, bleomycin, clofarabine, 5‐fluorouracil, vincristine, or dactinomycin. The AC and control groups did not differ by age (*P*=0.83) or sex (*P*=0.81) at study entry.

**Table 1:** Characteristics of the Study Population

	Anthracycline (AC), n=24				Controls (CTRL), n=9	*P* for AC vs CTRL
	Acute cTnT Elevation^a^	Chronic cTnT Elevation^b^	No cTnT Elevation^c^	*P* Across AC Groups		
No. of patients	7	8	9	···	9	···
Median age [range in y]	4 [0.5–16]	10.5 [3–16]	13 [7–16]	*P*=0.09	14 [1.8–18]	*P*=1.0
No. female (%)	4 (57)	1 (12)	2 (22)	*P*=0.20	2 (23%)	*P*=1.0
Diagnoses (n)	AML (6), NBL	Sarcoma (4), HL (2), ALL, APL	Sarcoma (3), NHL, HL (3), AML (2)	···	GCT (4), HBL, LCH, MED, NPC, RMS	···
Cumulative AC dose:^d^ median [range in mg/m2]	442 [75–442]	375 [75–600]	300 [75–450]	*P*=0.25	···	···
Interim AC dose:^e^ median [range in mg/m2]	192 [75–192]	67.5 [25–75]	60 [25–192]	*P*<0.01	···	···
No. receiving dexrazoxane (%)	3 (43)	2 (25)	3 (33)	*P*=0.87	···	···
No. with SF reduction ≥20% from AC naïve baseline (%)^f^	2 (28)	2 (25)	1 (11)	*P*=0.69	···	···
% SF reduction:^f^ median [range]	8 [3–35]	11 [0–26]	0 [0–24]	*P*=0.30	···	···
Peak hs‐cTnT: median [range in ng/L]	32 [20–149]	30 [14–57]	9 [0–12]	*P*<0.01	6 [0–10]	*P*<0.01
Max hs‐cTnT increase from baseline: median fold‐change [range]	2.7 [1.6–6.4]	1.1 [0.8–1.4]	1.1 [0.7–1.5]	*P*<0.01	1 [0.8–1.4]	*P*=0.49

ALL indicates acute lymphoid leukemia; AML, acute myeloid leukemia; APL, acute promyelocytic leukemia; GCT, germ cell tumor; HBL, hepatoblastoma; HL, Hodgkin's lymphoma; hs‐cTnT, high‐sensitivity cardiac troponin T; LCH, Langerhans histiocytosis; MED, medulloblastoma; NBL, neuroblastoma; NHL, non‐Hodgkin's lymphoma; Max, maximum; NPC, nasopharyngeal carcinoma; RMS, rhabdomyosarcoma.

a Acute cTnT elevation: hs‐cTnT ≥14 ng/L at any time point, plus ≥5 ng/L rise in hs‐cTnT between the precycle baseline (0 hour) and postcycle hs‐cTnT (6, 12, or 24 hours).

b Chronic cTnT elevation: hs‐cTnT ≥14 ng/L at any time point, but <5 ng/L rise in hs‐cTnT between the precycle baseline (0 hour) and postcycle hs‐cTnT (6, 12, or 24 hours).

c No cTnT elevation: hs‐cTnT <14 ng/L at all time points.

d Cumulative anthracycline dose=total anthracycline dose given before 6, 12, or 24 hours postanthracycline sample collection.

e Interim anthracycline dose=dose around which the pre‐ (0 hour) and postplasma (6, 12, and 24 hours) samples were collected. Anthracycline doses were converted to the equivalent doxorubicin dose: daunorubicin×0.83, epirubicin×0.67, idarubicin×5, and mitoxantrone×4.

f SF: shortening fraction; echocardiograms were obtained in patients receiving anthracyclines, before any anthracycline exposure and 1 to 3 months following the final anthracycline dose.

Of the 24 AC patients analyzed, 15 had hs‐cTnT concentrations ≥14 ng/L following the cycle dose(s) of AC (cTn(+) AC). Of these, 8 patients had troponin ≥14 ng/L at baseline, preceding this cycle of AC (mean cTnT: 23±SD 4.1 ng/L; Figure 1). Seven of the 15 hs‐cTnT‐positive patients had an acute hs‐cTnT rise of ≥5 ng/L from baseline. Nine patients had no hs‐cTnT elevations at any time point. Within the AC group, the patients with acute elevations were similar in age, sex, and cumulative AC dose compared to those with chronically elevated or normal hs‐cTnT (Table). However, the patients with acute elevations received a higher interim dose of AC than those with normal or chronically elevated hs‐cTnT (*P*=0.004).

### Evaluation of Plasma MicroRNA Dysregulation Over Time by Chemotherapy Group

**Figure 2:**
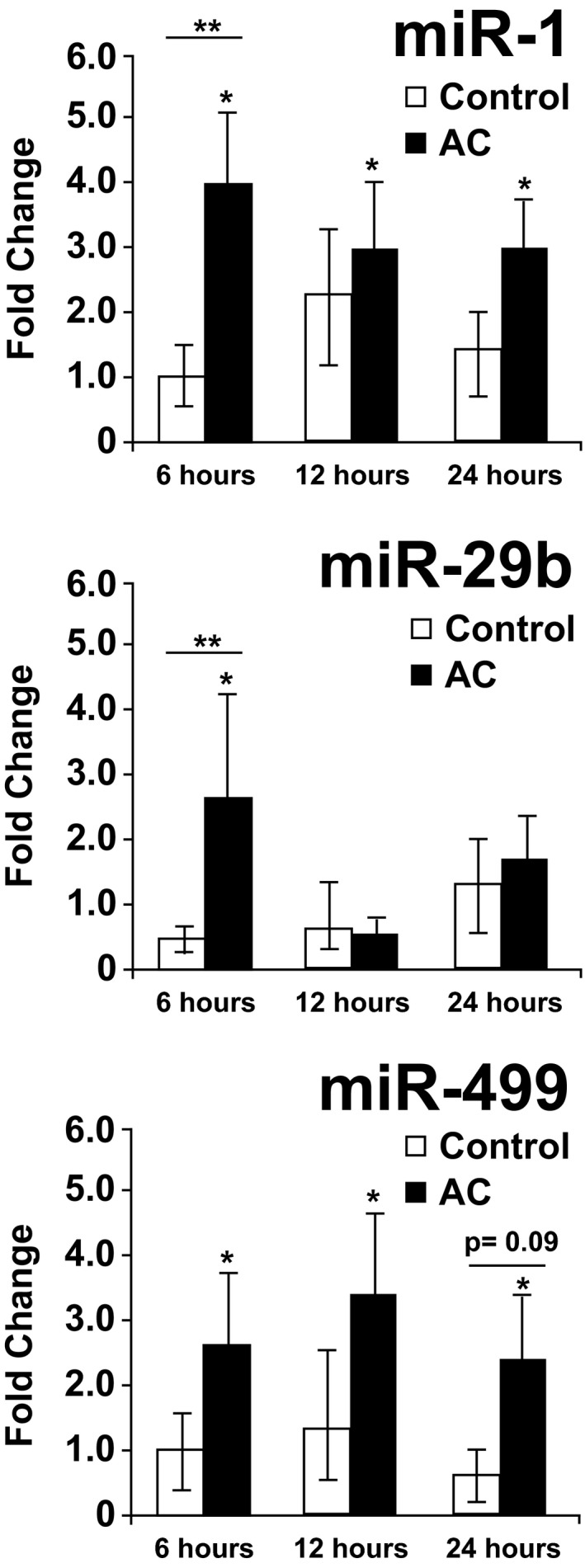
MicroRNA (miRNA) upregulation from baseline postanthracycline versus noncardiotoxic chemotherapy. Normalized miR‐1, ‐29b, and ‐499 were significantly upregulated at multiple time points following the cycle anthracycline (AC; n=24), while unchanged in controls (n=9) receiving noncardiotoxic chemotherapy. *P<0.05 within‐group change in miRNA postchemo (from baseline). **P<0.05 between group (AC vs control) difference in miRNA regulation postchemo.

Greater overall chemotherapy‐induced dysregulation was observed in the 24 candidate plasma miRNAs in patients receiving AC chemotherapy compared with those receiving noncardiotoxic chemotherapy (MANOVA at 24 hours, *P*=0.024). Change in plasma expression from baseline of 2 specific miRNAs differed significantly between AC and controls (*P* value for anthracycline versus control at 6 hours, <0.05; Table S3). Plasma miR‐1 was significantly upregulated 6, 12, and 24 hours post‐AC, compared with controls in whom it was unchanged at all time points (Figure 2; Table S3). Plasma miR‐29b demonstrated 2.7‐fold upregulation 6 hours post‐AC, while unchanged in controls (Figure 2). Additionally, miR‐499 was significantly upregulated by >2‐fold 6, 12, and 24 hours post‐AC compared with controls in whom it was unchanged and showed a trend toward differential regulation between treatment groups at 24 hours (*P*=0.09; Figure 2; Table S3).

### Relationship Between Plasma miR‐29b and ‐499 Expression and Anthracycline Dose

When comparing miRNA expression and AC dose, a significant relationship was identified between 6‐hour miR‐29b, 6‐hour miR‐499, and 24‐hour miR‐499 expression and interim AC dose, that is, higher miRNA expression with increasing AC dose for the cycle around which the plasma was collected (parameter estimates for miRNA ΔCt: 6‐hour miR‐29b=−0.017; *P*=0.020; 6‐hour miR‐499=−0.018; *P*=0.004; 24‐hour miR‐499=−0.028; *P*<0.001). Additionally, there was a significant relationship between 6‐ and 24‐hour miR‐499 and cumulative AC dose (estimate 6‐hour=−0.0065; *P*=0.006; 24‐hour=−0.0084; *P*=0.010). Finally, when controlling for concomitant cardioprotective therapy with dexrazoxane, a significant relationship between 6‐hour miR‐29b and cumulative anthracycline dose was observed (parameter estimate=−0.0387; *P*=0.033), whereas the relationship between 24‐hour miR‐499 and cumulative dose was no longer significant.

### Evaluation of Plasma MicroRNA Expression by Troponin Concentration

**Figure 3:**
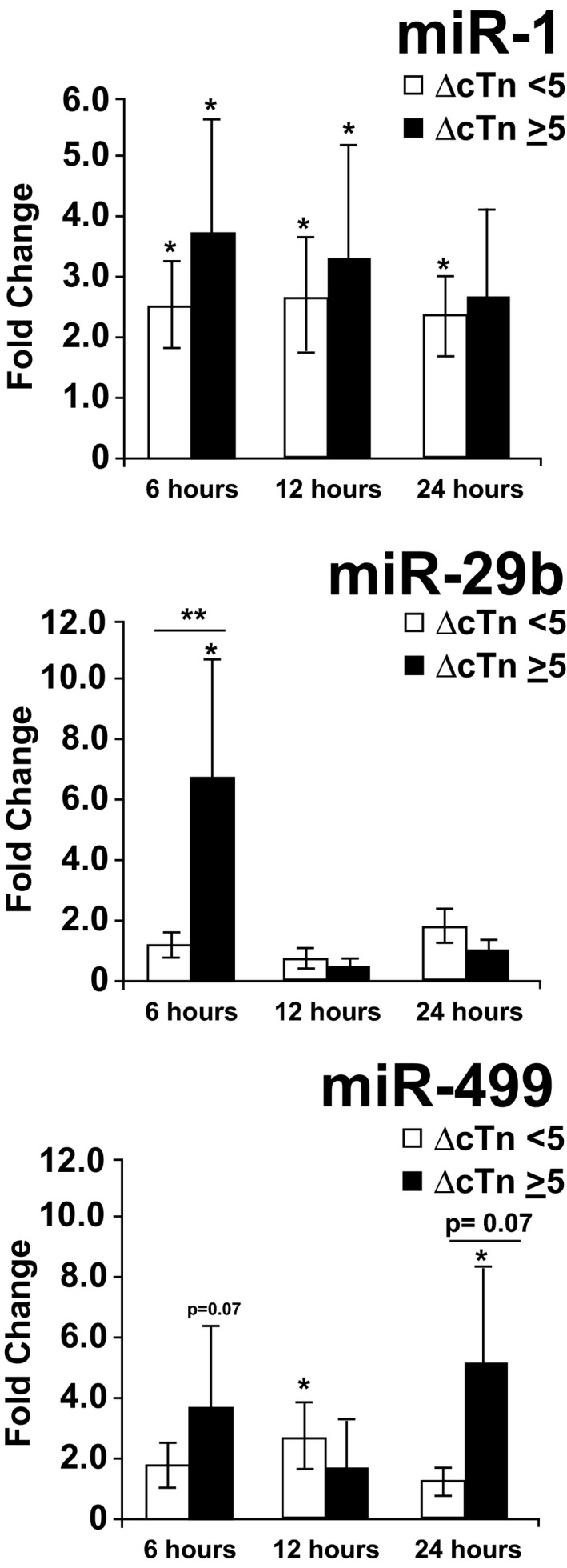
miRNA upregulation from baseline postchemotherapy by troponin group. Patients with ≥5‐ng/L postanthracycline rise in troponin from precycle baseline (ΔcTn ≥5; n=7) demonstrated upregulation of miR‐29b 6 hours following anthracycline chemotherapy when compared with patients with stable or negative troponin following anthracycline or noncardiotoxic chemotherapy (ΔcTn <5; n=26; between group, *P*=0.013). Whereas miR‐499 demonstrated a trend toward differential regulation between troponin groups, this difference was nonsignificant (*P*=0.07). Plasma miR‐1 was similarly upregulated across troponin groups. *P<0.05 within‐group change in miRNA postchemo (from precycle baseline). **P<0.05 between‐group (ΔcTn ≥5 vs cTn <5) difference in miRNA regulation postchemo. AC indicates anthracycline; cTn, cardiac troponin; miRNA, microRNA.

Further analysis of chemotherapy‐induced plasma miR‐1, ‐29b, and ‐499 regulation revealed significant differences between patients with and without evidence of acute cardiac injury following chemotherapy (hs‐cTnT increase ≥5 ng/L from baseline). Patients with acute AC‐induced hs‐cTnT elevations had significant upregulation in miR‐29b at 6 hours (6.6‐fold; *P*<0.01) and miR‐499 at 24 hours (5.2‐fold; *P*=0.03) compared with AC or control patients with chronically elevated or normal hs‐cTnT (Figure 3; between group *P*=0.01 for miR‐29b and *P*=0.07 for miR‐499). Plasma miR‐1 was similarly upregulated over time regardless of hs‐cTnT concentrations.

**Figure 4:**
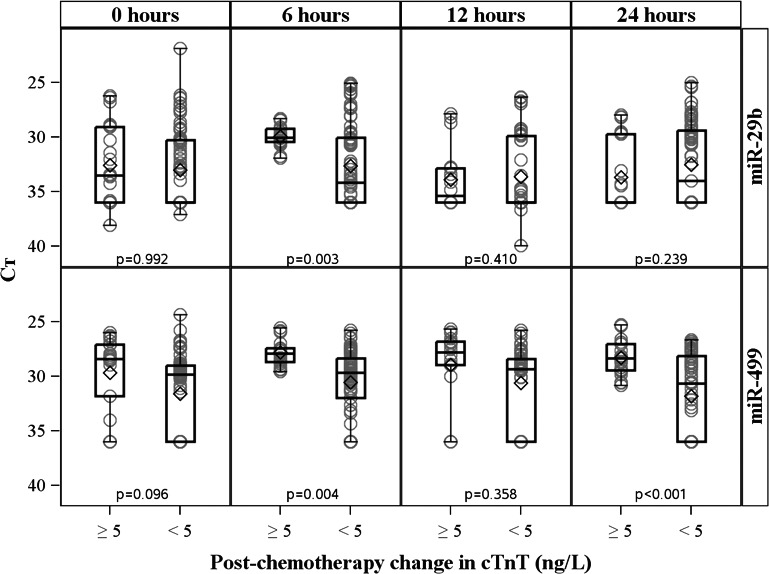
Normalized miRNA expression at each time point by troponin group. Normalized plasma miR‐29b (top row) and miR‐499 (bottom row) expression (ΔCt [cycle threshold]) of patients with acute troponin elevations (rise in cTnT of ≥5 ng/L; n=7) compared with that of patients with stable or negative troponin postchemotherapy (n=26). Patients with troponin evidence of acute cardiac injury demonstrated higher plasma expression of miR‐29b at 6 hours and miR‐499 at 6 and 24 hours post‐AC compared with those with chronically elevated or normal hs‐cTnT. Mean Ct difference expressed on the *y*‐axis is the cycle threshold of each candidate miRNA normalized to the aggregate Ct of endogenous controls (miR‐484 and miR‐140). Each box represents the interquartile range of miRNA expression (25th–75th percentile), with the horizontal line representing the median and the diamond representing the mean. hs‐cTnT, high‐sensitivity cardiac troponin T; miRNA, microRNA.

Similar results were observed when normalized plasma miR‐29b and miR‐499 expression (ΔCt) was compared across groups at each pre‐ and postchemotherapy time point. Patients with acute cardiac injury demonstrated significantly higher plasma expression of miR‐29b at 6 hours and miR‐499 at 6 and 24 hours compared with those with chronically elevated or normal hs‐cTnT (Figure 4).

Six‐hour miR‐29b, 6‐hour miR‐499, and 24‐hour miR‐499 individually moderately discriminated patients with evidence of acute cardiac injury post‐AC (hs‐cTnT rise, ≥5 ng/L; area under the curve [AUC], 0.75, 0.82, and 0.80, respectively). Discrimination for acute cardiac injury improved when using an aggregate of 6‐hour miR‐29b and miR‐499 (AUC, 0.90). Individual or aggregate 12‐hour miR‐29b or miR‐499 levels did not accurately discriminate patients with evidence of acute cardiac injury (AUC, 0.61, 0.68, 0.63 for miR‐29b, miR‐499, and miR‐29b/‐499, respectively).

## Discussion

This study is the first to analyze circulating miRNAs in the setting of AC‐induced cardiotoxicity. Although these findings are preliminary given the exploratory nature and small sample size, there were several key findings that support further exploration of miRNAs as clinically relevant markers of AC‐induced cardiotoxicity. First, overall dysregulation of a panel of candidate plasma miRNAs with cardiac relevance was greater following AC compared with noncardiotoxic chemotherapy. Second, plasma miR‐29b and miR‐499 were upregulated in children with acute AC‐induced cardiac injury, as evidenced by rising troponin concentrations post‐AC. Third, post‐AC miR‐29b and miR‐499 expression significantly correlated with cumulative AC dose, an established predictor of cardiotoxicity risk. Finally, plasma miR‐29b and miR‐499 expression levels 6 to 24 hours following AC provided moderate discrimination of patients with and without acute, cardiac injury. Together, these findings indicate that miRNAs may have a role in detecting subclinical cardiotoxicity.

miRNAs are critical modulators of cardiovascular development and disease, wherein miRNA‐guided expression of downstream targets governs the process of regeneration, repair, or pathological remodeling.^11^ Specific profiles of miRNA expression have been identified in numerous cardiovascular disease states, wherein circulating expression profiles may provide insight into underlying injury or remodeling occurring in the cardiac tissue.^24^, ^25^ These circulating miRNA profiles are not only windows into the molecular landscape of heart disease, but offer the potential for novel therapeutics aimed at the modulation of miRNA and downstream target expression.^15^ Additionally, accumulating evidence supports the value of miRNAs as robust biomarkers of cardiovascular disease.^13^, ^15^ Relevant to our findings, miR‐499 is a muscle‐specific, cardiomyocyte‐enriched miRNA that has been shown to have high sensitivity and specificity for detecting myocardial ischemia in previous reports.^15^, ^26^, ^27^ Plasma miR‐499 upregulation postinfarction has been observed even earlier than hs‐cTnT and thus may provide superior early sensitivity than troponins.^28^ Additionally, higher plasma miR‐499 expression following acute myocardial infarction has been correlated with increased risk for heart failure or mortality, indicating that miRNA profiles in the setting of acute injury may have important prognostic implications.^27^ The protective role of miR‐499 against oxidative‐stress–induced apoptosis following acute myocardial infarction^29^ has important relevance to AC‐induced cardiotoxicity given that the most accepted mechanism of AC‐induced cardiotoxicity is through the generation of reactive oxygen species and mitochondrial dysfunction within the cardiomyocyte.^30^ Interestingly, in our small cohort, the protective role of dexrazoxane against AC‐induced oxidative stress abrogated the relationship between cumulative anthracycline dose and miR‐499 elevations, perhaps indicating that dexrazoxane was protective against oxidative‐stress–related miR‐499 elevations, even at high anthracycline doses.

The miR‐29 family members are potent inhibitors of cardiac fibrosis and play a key role in cardiac remodeling following cardiomyocyte injury.^31^ Plasma miR‐29a upregulation has been reported following myocardial injury, where the degree of mR‐29a upregulation was associated with the extent of late remodeling post‐acute myocardial infarction.^24^ miR‐29b, reported herein, targets many genes involved in the extracellular matrix (ECM), such as fibronectin, collagen, and matrix metalloproteinases. Given the critical role of early and late ECM remodeling in response to AC‐induced cardiotoxicity,^32^, ^33^ the upregulation of plasma miR‐29b in this study has intriguing mechanistic relevance and may reflect early remodeling in response to AC‐induced cardiac injury. Furthermore, modulation of miR‐29 in various settings has successfully reduced maladaptive remodeling postinjury and allowed a more‐efficient ECM remodeling response.^31^, ^34^ With ongoing progress toward miRNA‐directed therapy, the elucidation of the role of miR‐29b in AC‐induced cardiotoxicity could reveal an important therapeutic target that might allow prevention of AC‐induced remodeling leading to preserved cardiac function.

This study has several notable limitations. First, the miRNA expression patterns identified in this study are based on a small, heterogeneous group of patients who received varying anthracycline regimens for a variety of malignancies. Additionally, the disproportionate sample sizes between the control group and AC group poses a significant statistical limitation. However, despite this heterogeneity and small sample size, miRNA expression correlated with multiple indicators/predictors of cardiotoxicity, including AC dose and hs‐cTnT. Although troponin elevation has not consistently been shown to correlate with late cardiac outcomes in AC‐exposed childhood cancer survivors, it is clearly a reflection of cardiomyocyte injury.^35^ Thus, use of high‐sensitivity troponin changes as a cardiotoxicity correlate for this preliminary miRNA analysis is reasonable. Unfortunately, changes in echocardiogram parameters are typically a late finding in pediatric cardiotoxicity occurring many years from AC exposure. Thus, more‐definitive imaging correlates on which to compare miRNA expression were not feasible. The candidate approach to miRNA assessment in the setting of AC‐induced cardiotoxicity may have limited our ability to identify miRNAs with important diagnostic or mechanistic implications. However, the 2 miRNAs found to have the most significant plasma dysregulation in patients with AC‐induced cardiac injury are promising, particularly given their intriguing mechanistic relevance to that implicated in cardiotoxicity.

A final limitation of this and other circulating miRNA profiling studies is that the etiology and mechanistic significance of plasma miRNA dysregulation is unknown. Although it is clear that many miRNAs (eg, miRs‐499, ‐208, ‐133, and ‐1) are released into the plasma following cellular injury (as demonstrated after myocardial infarction), miRNAs are also exported from intact cells by being bound to stabilizing proteins or packaged within exosomes or microvesicles. Given evidence of troponin elevations following AC exposure, plasma miRNA expression described herein may, in part, reflect miRNA release following myocardial injury. Given the small sample size of this study, it cannot be definitively concluded that the other candidate miRNAs previously associated with cardiomyocyte injury (eg, in the setting of acute myocardial infarction, such as miR‐133a)^15^ are not associated with AC‐induced cardiomyocyte injury. However, the lack of relationship observed in this study between these cardiac miRNAs and AC might reflect the unique molecular functions of each individual miRNA, which might not be significantly involved in the heart's response to AC. Further work is warranted to determine the source and mechanistic implications of miR‐29b and 499 elevations in the plasma.

In conclusion, we report that plasma miR‐29b and ‐499 elevations occur in the acute setting following AC exposure and may be useful in detecting cardiomyocyte injury. Further work is warranted to evaluate the mechanistic role of these miRNAs in AC‐induced cardiac injury and determine whether these miRNAs are useful for identifying patients at high risk of developing AC‐induced cardiomyopathy.

## Sources of Funding

Research reported in this publication was supported by the National Center for Advancing Translational Sciences of the National Institutes of Health under Award Number UL1TR001105, as well as an NIH Training in Cardiovascular Research Grant (Award Number T32HL007360‐35). Additional research funding was provided by Conquer Cancer & Strike3 Foundations under an American Society of Clinical Oncology (ASCO) Young Investigator Award (granted to Leger) and the Children's Cancer Fund of Dallas (granted to Leger), and a NIH R01 Research Grant (R01 HL102478 granted to Mammen). The content is solely the responsibility of the authors and does not necessarily represent the official views of the ASCO or NIH.

## Disclosures

The following authors have relationships with industry to disclose: Dr DeLemos: Amgen (advisory role, research funding provided), Roche Diagnostics (advisory role, research funding provided), and Abbott Diagnostics (research funding provided); Dr Mammen: CareDx Inc (advisory role, speaker's bureau, and research funding/honoraria/travel accommodations provided), HeartWare (advisory role, honoraria/travel accommodations provided), PhaseBio (advisory role, honoraria/travel accommodations provided), and California Institute for Regenerative Medicine (advisory role); Nielson: United Therapeutics (advisory role, speaker's bureau, and travel accommodations provided). Additional disclaimer: Roche Diagnostics provided hs‐cTnT measurements, however had no role in the study.

## Supporting information


**Data S1.** Supplemental methods.
**Table S1.** Selection of Candidate (A) and Control (B) miRNAs Based on Published Literature
**Table S2.** Stability Score of Potential Endogenous Controls, as Determined by Normfinder Algorithm [35]
**Table S3.** Dysregulation of Candidate miRNAs Postanthracycline or Noncardiotoxic Chemotherapy
**Figure S1.** Troponin trajectory over time by treatment group.
