# Supplementary material for: Circulating microRNAs: Potential Markers of Cardiotoxicity in Children and Young Adults Treated With Anthracycline Chemotherapy
Source: J Am Heart Assoc. 2017 Apr 4;6(4):e004653. doi: 10.1161/JAHA.116.004653 (PMC5532993; doi:10.1161/JAHA.116.004653)
Supplement: Supplementary file 1 — Data S1. Supplemental methods. Table S1. Selection of Candidate (A) and Control (B) miRNAs Based on Published Literature Table S2. Stability Score of Potential Endogenous Controls, as Determined by Normfinder Algorithm [35] Table S3. Dysregulation of Candidate miRNAs Postanthracycline or Noncardiotoxic Chemotherapy Figure S1. Troponin trajectory over time by treatment group. [file JAH3-6-e004653-s001.pdf]

SUPPLEMENTAL MATERIAL

Data S1.

SUPPLEMENTAL METHODS:

Sample processing and RNA isolation

Plasma samples were collected following patient enrollment into an Institutional Review Board approved research protocol (University of Texas Southwestern IRB). Study investigators obtained informed consent from each participant or each participant's guardian. Plasma samples were collected either via peripheral venipuncture or from the patient's central line after sufficient waste was withdrawn to avoid heparin contamination. Within one hour of collection, samples were processed via refrigerated centrifuge at 2500 x G for 15 minutes. Plasma used for RNA isolation underwent a second high speed spin at 14,000 x G for 10 minutes to decrease platelet and particle contamination. RNA was isolated using Qiagen's miRNeasy serum/ plasma microRNA isolation kit (Qiagen, cat no. 217184) according to the manufacturers protocol with the following modifications: (1) 750uL Qiazol Lysis reagent added to 200uL plasma (vs. the 5:1 ratio recommended in the protocol) (2) 1.25µl 0.8 µg/µl bacteriophage MS2 RNA added upon addition of Qiazol solution in order to improve RNA yield (3) Wash with RPE repeated x 3 (in place of ETOH step). (3) RNA eluted in 20uL of nuclease free water.

RNA Quantification & Processing of miRNA Expression Data

Custom TaqMan® Array MicroRNA Cards (Applied Biosystems; Product# 4346799, Format 32) were designed to assay 24 candidate miRNAs and 7 miRNAs for quality assessment and normalization. Candidate miRNAs were selected based on their demonstrated roles as biomarkers or mediators of cardiovascular disease (Table S1) and include let-7g, miRs-1, 133a, 133b, 145, 146a, 195, 208a, 208b, 21, 210, 214, 215, 216b, 29a, 29b, 29c, 30a-5p, 34c, 361, 367, 423-3p, 423-5p, and 499-5p. A group

of candidate endogenous control miRNAs, including miRs-103, 140, 191-5p, 484, U6, and synthetic cel-miR-39, were also selected based on reports of successful normalization achieved using these miRNAs.[1-4] Additionally miR-451 and miR-23a were assayed as a quality control measure to assess sample hemolysis.[5]

Reverse transcription utilizing the Taqman custom RT primer pool was performed using the Taqman miRNA reverse transcription kit. Reaction volumes were modified based on the Taqman technical note entitled “Optimized protocols for human or rodent microRNA profiling with precious samples” (Product bulletin 4478705), including the following volumes: 3uL undiluted RNA, 4uL custom primer pool, 0.2uL dNTP, 2uL reverse transcriptase, 1uL 10X buffer, and 0.13uL RNAse inhibitor for a total volume of 10.33uL. Pre-amplification was performed utilizing the Taqman PreAmp Mastermix and custom primer pool, with volumes and number of amplification cycles modified based on the Taqman technical note (Product bulletin 4478705). The reaction included 5uL cDNA, 3.75uL water, and 3.75uL custom PreAmp primer pool and underwent 14 amplification cycles. Finally, 2.25uL of the undiluted preamp product was combined with 112.5uL Taq PCR mastermix and 110.25uL nuclease free water (total volume of 225uL for 2 fill ports) and pipetted onto the custom miRNA TLDA cards. Real time analysis was performed per the manufacturer’s protocol using an Applied Biosystems 7900HT Real time PCR System.

Cycle threshold data for all samples were imported into OmicsOffice Statminer software program’s real time qPCR analysis feature. Undetermined cycle threshold values were set to 36, the detection limit. Candidate endogenous control miRNAs were evaluated using the normfinder algorithm aimed at identifying the miRNA(s) with the lowest variation within groups (AC vs. control). The aggregate of miRs-140 and -484 was selected as the endogenous control for this study based on its lowest stability value (Table S2).

Plasma microRNA Normalization

Currently there is a lack of consensus regarding circulating miRNA normalization. Although many groups are normalizing serum and plasma miRNA expression to a spiked in synthetic miRNA, this does not account for the effects of plasma processing and other pre-analytic variables on circulating miRNA measurement.[6] Given the high content of many intracellular miRNAs relative to that of plasma, hemolysis and platelet contamination of plasma can introduce significant miRNA variability from one sample to the next.[6, 7] We employed several strategies to overcome these technical challenges, including an additional high-speed centrifugation step to minimize platelet contamination and assessing the degree of RBC-derived miRNA contamination due to hemolysis.[7, 8] Fortunately, tissue specific miRNAs with little or no expression in circulating blood cells (including miR-499 from this study) are relatively unaffected by these processing variables. However, miR-29b and other miRNAs examined herein are highly expressed in blood cells, thus may be sensitive to processing conditions and inadequately normalized by the synthetic spike-in strategy.[9] For this reason, endogenous miRNA controls, chosen based on stability score (Table S2), were used for candidate miRNA normalization in this study.

High-sensitivity Troponin Assay

cTnT was measured in sample aliquots previously stored at −80°C using a highly sensitive automated immunoassay (Troponin T hs STAT, Elecsys-2010, Roche Diagnostics, Indianapolis, Indiana), with a limit of detection of 5 ng/l and a limit of blank of 3 ng/l. The lowest hs-cTnT concentration that can be measured with a coefficient of variation ≤ 10% with this assay is 13 ng/l.[10]

Statistical Analysis

Plasma miRNA dysregulation over time by chemotherapy group. For each patient and candidate miRNA, 3 cycle threshold measurements were made at each time point. Linear mixed effects regression models for each target miRNA were used to estimate normalized mean cycle threshold values over time by chemotherapy group and allow comparison across groups. The models included fixed effects of group (anthracycline versus non-cardiotoxic chemotherapy), time (0, 6, 12, and 24 hours), condition (target miRNA vs. endogenous control miRs-140 and -484), and all 2- and 3- factor cross-terms. The models included random intercept, time, condition, and time × condition by patient effects to account for correlated cycle threshold levels, changes over time, differences between conditions, and differences between changes over time by condition within patients, respectively. The models allowed the residual variation in repeated measurements across time to differ by condition within patients. Normalized cycle threshold by group and time was estimated as the difference between the estimated mean target cycle threshold by group and time and the arithmetic average of the corresponding mean cycle thresholds of the two endogenous controls.

Plasma miRNA dysregulation over time by troponin group. Similar mixed effects regression models were constructed to assess miRNA expression over time by troponin group. These models were identical to that used for chemotherapy group, except that troponin group (≥5 versus <5ng/L post-chemotherapy change in troponin) was used in place of chemotherapy group. While population studies of normal adults support a threshold of 14ng/L for cTnT concentrations above the 99th percentile, more recent data supports the use of change in troponin over time to discriminate patients with and without acute cardiomyocyte injury.[11-14] The threshold for acute cardiomyocyte injury of 5ng/L rise 6-24

hours post chemotherapy is consistent with the clinically meaningful rise over a similar time period in the setting of myocardial infarction.[13, 14]

Sample size

A priori, we targeted recruitment of 34 patients (24 anthracycline-exposed; 10 controls), which would provide 80% power to detect a 2 fold-mean group change in miRNA expression from baseline between groups, with an alpha of 0.05. Variability in miRNA expression was calculated based on pilot data in the Mammen Laboratory revealing cycle threshold variability of 1.5-2.5 standard deviations, and we made a conservative estimate of sample size (n=30) based on SD of 2.5. Reported p-values were not adjusted for multiple comparisons. All analyses were programmed in SAS/STAT® version 9.4 (SAS Institute, Cary, NC, USA).

Table S1. Selection of candidate (A) and control (B) miRNAs based on published literature

(A) Candidate miRNAs	
Biomarker of cardiomyocyte (CM) injury post-myocardial infarction and/or mechanistically involved in cardiomyocyte proliferation & survival	miR-1 [15, 16]<br>miR-133a/b [16, 17]<br>miR-195 [18]<br>miR-208a/b [19, 20]<br>miR-21 [21]<br>miR-30a-5p [22]<br>miR-499-5p [19, 23]
Biomarker of heart failure	miR-423-5p [24]
Mediator/ Biomarker of Cardiac Fibrosis	miR-29a/b/c [25, 26]
Mediator of ROS signaling within the cardiomyocyte	miR-145 [27]<br>miR-210 [28]<br>miR-214 [29]<br>miR-361 [30]
Mediators of (or dysregulated in) doxorubicin-induced cardiac injury models	miR-21 [31]<br>miR-34c [32]<br>miR-146a [33]<br>miR-215 [32]<br>miR-216b [32]<br>miR-367 [32]<br>miR-423-3p*<br>let-7g [34]
**(**B) Endogenous Control miRNAs	
Role in normalizing plasma miRNAs	miR-103 [3]<br>miR-140†<br>miR-191-5p [1]<br>miR-484 [1]<br>cel-miR-39 [2]
miRNAs used to assess degree of hemolysis	miR-23a [5]<br>miR-451 [5]

[ ] Denotes manuscript reference

*Selected based on differential regulation observed in pilot data involving plasma miRNA array profiling (performed by Exiqon) of patients with chronic anthracycline cardiomyopathy compared to controls.

†Selected based on a low stability score in pilot data involving plasma miRNA array profiling (performed by Exiqon) on samples drawn and processed in the same manner by the same institution

Table S2. Stability score of potential endogenous controls, as determined by normfinder algorithm[35]

Endogenous Control(s)	Stability Score
hsa-miR-140 + miR-484	0.057
hsa-miR-484	0.058
miR-103 + miR-484	0.092
miR-140	0.097
miR-191 + miR-484	0.098
miR-103+ miR-140	0.101
miR-140+ miR-191	0.106
miR-103 + miR-191	0.128
cel-miR-39 + miR-484	0.166
cel-miR-39 + miR-140	0.171
miR-103	0.174
cel-miR-39 + miR-103	0.185
miR-191	0.186
cel-miR-39 + miR-191	0.189
cel-miR-39	0.326

Table S3. Dysregulation of candidate miRNAs post-anthracycline or non-cardiotoxic chemotherapy

		Anthracycline Group				Non-Cardiotoxic Chemotherapy Group				Between Group Difference
		Fold Change	Lower 95% CL	Upper 95% CL	Pr > |t|	Fold Change	Lower 95% CL	Upper 95% CL	Pr > |t|	Pr > |t|
**miRNA**	**Time (hr)**									
**let-7g**	**6**	1.3	0.9	1.7	0.108	1.0	0.6	1.6	0.841	0.312
	**12**	1.3	0.9	1.8	0.158	1.1	0.7	1.8	0.722	0.609
	**24**	1.1	0.8	1.4	0.675	0.7	0.4	1.1	0.088	0.095
**miR-1**	**6**	**3.9**	**2.2**	**7.0**	**<0.001**	1.0	0.4	2.5	0.973	**0.015**
	**12**	3.0	1.6	5.9	0.001	2.2	0.8	5.8	0.113	0.598
	**24**	3.0	1.7	5.3	<0.001	1.4	0.5	3.6	0.486	0.178
**miR-133a**	**6**	1.1	0.8	1.5	0.441	0.9	0.5	1.5	0.725	0.482
	**12**	1.0	0.7	1.4	0.851	1.1	0.6	1.8	0.800	0.752
	**24**	1.2	0.9	1.7	0.208	1.0	0.6	1.7	0.944	0.544
**miR-133b**	**6**	1.4	0.8	2.4	0.210	2.1	0.9	5.1	0.108	0.472
	**12**	1.3	0.7	2.4	0.487	0.5	0.2	1.4	0.198	0.146
	**24**	0.9	0.5	1.6	0.797	0.9	0.4	2.1	0.776	0.916
**miR-145**	**6**	1.2	0.9	1.6	0.288	1.5	0.9	2.6	0.096	0.384
	**12**	1.1	0.8	1.6	0.616	1.3	0.8	2.2	0.307	0.578
	**24**	1.0	0.8	1.4	0.852	1.1	0.7	1.8	0.678	0.799
**miR-146a**	**6**	1.0	0.7	1.3	0.758	1.2	0.8	1.8	0.499	0.461
	**12**	0.9	0.6	1.2	0.404	1.3	0.8	2.0	0.299	0.184
	**24**	1.1	0.8	1.4	0.719	1.5	1.0	2.4	0.055	0.148
**miR-195**	**6**	1.4	1.1	1.7	< 0.001	1.2	0.9	1.6	0.252	0.430
	**12**	1.6	1.3	2.0	< 0.001	1.5	1.1	2.1	0.011	0.742
	**24**	1.7	1.4	2.1	< 0.001	1.3	0.9	1.7	0.160	0.099
**miR-21**	**6**	1.6	1.3	2.0	< 0.001	1.9	1.3	2.7	< 0.001	0.390
	**12**	1.6	1.2	2.1	< 0.001	2.0	1.4	2.8	< 0.001	0.365
	**24**	1.6	1.3	2.0	< 0.001	1.7	1.2	2.4	0.002	0.703

**miR-210**	**6**	1.3	1.1	1.5	< 0.001	1.3	1.0	1.6	0.050	0.870
	**12**	1.3	1.1	1.6	0.003	1.3	1.0	1.7	0.024	0.891
	**24**	1.4	1.2	1.6	< 0.001	1.4	1.1	1.8	0.004	0.806
**miR-214**	**6**	1.1	0.8	1.5	0.612	1.0	0.6	1.5	0.837	0.660
	**12**	1.5	1.1	2.2	0.019	1.2	0.7	1.9	0.551	0.391
	**24**	1.5	1.1	2.1	0.007	0.9	0.6	1.5	0.794	0.096
**miR-215**	**6**	1.1	0.8	1.5	0.676	1.0	0.6	1.7	0.952	0.868
	**12**	1.2	0.8	1.7	0.393	1.6	0.9	2.7	0.095	0.374
	**24**	1.6	1.2	2.3	0.003	0.9	0.5	1.5	0.694	0.059
**miR-216b**	**6**	0.6	0.3	1.2	0.157	0.4	0.1	1.3	0.126	0.571
	**12**	1.6	0.7	3.7	0.269	0.6	0.2	1.9	0.347	0.161
	**24**	0.6	0.3	1.3	0.227	0.5	0.2	1.7	0.264	0.755
**miR-23a**	**6**	1.3	1.0	1.7	0.041	1.6	1.0	2.4	0.048	0.538
	**12**	1.4	1.0	1.9	0.045	1.5	0.9	2.3	0.096	0.823
	**24**	1.5	1.1	2.0	0.004	1.7	1.1	2.7	0.019	0.627
**miR-29a**	**6**	1.3	1.0	1.6	0.025	1.3	0.9	1.8	0.120	0.878
	**12**	1.2	0.9	1.5	0.233	1.4	1.0	2.0	0.068	0.407
	**24**	1.1	0.9	1.4	0.343	1.2	0.8	1.7	0.320	0.731
**miR-29b**	**6**	**2.7**	**1.4**	**5.4**	**0.004**	0.4	0.1	1.1	0.084	**0.003**
	**12**	0.6	0.3	1.4	0.263	0.8	0.3	2.5	0.710	0.742
	**24**	1.8	0.9	3.6	0.092	1.3	0.4	3.8	0.666	0.599
**miR-29c**	**6**	1.4	1.1	1.7	0.002	1.4	1.0	2.0	0.034	0.882
	**12**	1.4	1.1	1.7	0.009	1.5	1.0	2.0	0.031	0.776
	**24**	1.4	1.1	1.7	0.002	1.3	0.9	1.8	0.122	0.752
**miR-30a**	**6**	1.2	1.0	1.5	0.026	1.3	1.0	1.8	0.045	0.585
	**12**	1.3	1.1	1.6	0.016	1.2	0.9	1.6	0.230	0.693
	**24**	1.4	1.2	1.7	< 0.001	1.4	1.0	1.8	0.037	0.810
**miR-34c**	**6**	1.3	0.8	2.0	0.268	1.1	0.6	2.3	0.726	0.779
	**12**	2.6	1.6	4.3	< 0.001	1.5	0.7	3.0	0.315	0.209
	**24**	1.9	1.3	3.0	0.003	1.0	0.5	2.1	0.920	0.139
**miR-361**	**6**	1.1	0.9	1.5	0.435	1.6	1.0	2.4	0.044	0.187

	**12**	1.1	0.8	1.5	0.447	1.4	0.9	2.2	0.153	0.456
	**24**	1.4	1.1	1.9	0.013	1.4	0.9	2.2	0.104	0.949
**miR-367**	**6**	1.1	0.8	1.6	0.521	2.1	1.1	3.9	0.018	0.091
	**12**	1.0	0.7	1.6	0.899	1.9	1.0	3.7	0.044	0.111
	**24**	1.3	0.9	1.9	0.181	1.1	0.6	2.0	0.824	0.604
**miR-423_3P**	**6**	1.0	0.8	1.2	0.907	1.4	0.9	2.0	0.104	0.148
	**12**	0.7	0.6	0.9	0.017	1.0	0.7	1.4	0.968	0.180
	**24**	0.9	0.7	1.1	0.212	1.3	0.9	1.9	0.155	0.063
**miR-423_5p**	**6**	1.1	0.9	1.3	0.407	1.5	1.1	2.1	0.008	0.065
	**12**	1.0	0.8	1.3	0.699	1.5	1.0	2.0	0.027	0.109
	**24**	1.1	0.9	1.3	0.421	1.3	1.0	1.9	0.064	0.248
**miR-499**	**6**	2.7	1.2	5.9	0.014	1.0	0.3	3.5	0.960	0.183
	**12**	3.3	1.3	8.1	0.011	1.5	0.4	5.6	0.542	0.345
	**24**	2.4	1.1	5.2	0.035	0.6	0.2	2.3	0.495	0.091

miR-208a and b not included because undetectable in essentially all patients

Bolded red miRNAs are those with between group (AC vs. C) change in cycle threshold $p<0.05$

Figure S1. Troponin trajectory over time by treatment group

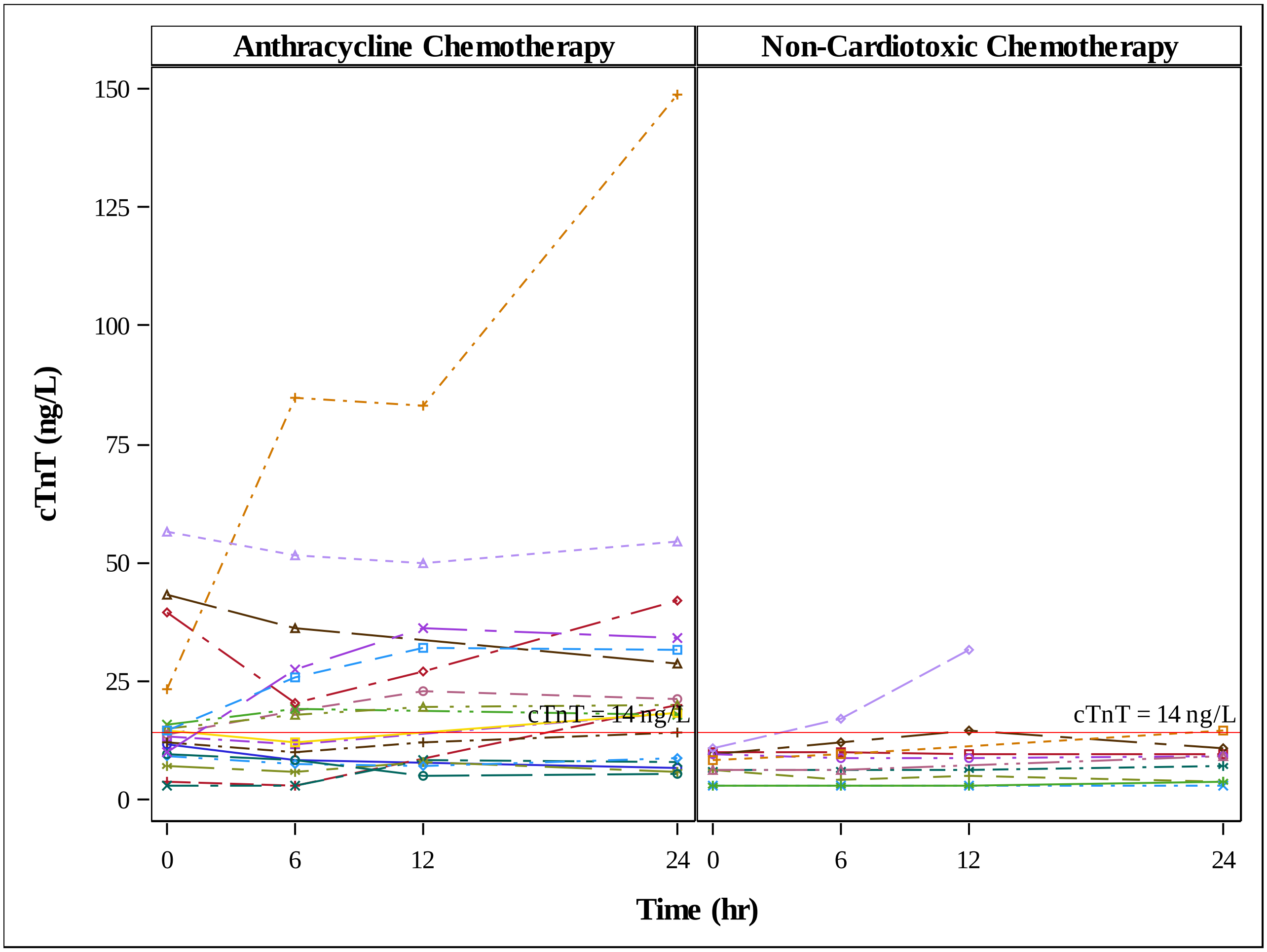

Each color and symbol represents an individual patient's plasma troponin prior to (0 hour) and following (6, 12, and 24 hours) a cycle of anthracycline or non-cardiotoxic chemotherapy. The dotted red line represents the standard threshold for a "positive troponin" at 14ng/L.

Supplemental References:

1. Hu Z, Dong J, Wang LE, Ma H, Liu J, Zhao Y, Tang J, Chen X, Dai J, Wei Q, Zhang C, Shen H. Serum microRNA profiling and breast cancer risk: the use of miR-484/191 as endogenous controls. *Carcinogenesis.* 2012; 33: p. 828-34.

2. Mitchell PS, Parkin RK, Kroh EM, Fritz BR, Wyman SK, Pogosova-Agadjanyan EL, Peterson A, Noteboom J, O'Briant KC, Allen A, Lin DW, Urban N, Drescher CW, Knudsen BS, Stirewalt DL, Gentleman R, Vessella RL, Nelson PS, Martin DB, Tewari M. Circulating microRNAs as stable blood-based markers for cancer detection. *Proc Natl Acad Sci U S A*. 2008; 105: p. 10513-8.

3. Wang Y, Tang N, Hui T, Wang S, Zeng X, Li H, Ma J. Identification of endogenous reference genes for RT-qPCR analysis of plasma microRNAs levels in rats with acetaminophen-induced hepatotoxicity. *J Appl Toxicol.* 2013; 33: p. 1330-6.

4. Robbins PF, Dudley ME, Wunderlich J, El-Gamil M, Li YF, Zhou J, Huang J, Powell DJ Jr, Rosenberg SA. Cutting edge: persistence of transferred lymphocyte clonotypes correlates with cancer regression in patients receiving cell transfer therapy. *J Immunol.* 2004;173: p. 7125-30.

5. Blondal T, Jensby Nielsen S, Baker A, Andreasen D, Mouritzen P, Wrang Teilum M, Dahlsveen IK. Assessing sample and miRNA profile quality in serum and plasma or other biofluids. *Methods.* 2013; 59: p. S1-6.

6. McDonald JS, Milosevic D, Reddi HV, Grebe SK, Algeciras-Schimnich A. Analysis of circulating microRNA: preanalytical and analytical challenges. *Clin Chem.* 2011; 57: p. 833-40.

7. Cheng HH, Yi HS, Kim Y, Kroh EM, Chien JW, Eaton KD, Goodman MT, Tait JF, Tewari M, Pritchard CC. Plasma processing conditions substantially influence circulating microRNA biomarker levels. *PLoS One.* 2013;8: p. e64795.

8. Kirschner MB, Edelman JJ, Kao SC, Vallely MP, van Zandwijk N, Reid G. The Impact of Hemolysis on Cell-Free microRNA Biomarkers. *Front Genet.* 2013; 4: p. 94.

9. Pritchard CC, Kroh E, Wood B, Arroyo JD, Dougherty KJ, Miyaji MM, Tait JF, Tewari M. Blood cell origin of circulating microRNAs: a cautionary note for cancer biomarker studies. *Cancer Prev Res (Phila).* 2012; 5: p. 492-7.

10. Powell DJ Jr, Dudley ME, Hogan KA, Wunderlich JR, Rosenberg SA. Adoptive transfer of vaccine-induced peripheral blood mononuclear cells to patients with metastatic melanoma following lymphodepletion. *J Immunol.* 2006; 177: p. 6527-39.

11. Giannitsis E, Kurz K, Hallermayer K, Jarausch J, Jaffe AS, Katus HA. Analytical validation of a high-sensitivity cardiac troponin T assay. *Clin Chem.* 2010; 56: p. 254-61.

12. Saenger AK, Beyrau R, Braun S, Cooray R, Dolci A, Freidank H, Giannitsis E, Gustafson S, Handy B, Katus H, Melanson SE, Panteghini M, Venge P, Zorn M, Jarolim P, Bruton D, Jarausch J, Jaffe AS. Multicenter analytical evaluation of a high-sensitivity troponin T assay. *Clin Chem Acta.* 2011; 412: p. 748-54.

13. Mueller M, Biener M, Vafaie M, Doerr S, Keller T, Blankenberg S, Katus HA, Giannitsis E. Absolute and relative kinetic changes of high-sensitivity cardiac troponin T in acute coronary syndrome and in patients with increased troponin in the absence of acute coronary syndrome. *Clin Chem.* 2012;58: p. 209-18.

14. Simpson AJ, Potter JM, Koerbin G, Oakman C, Cullen L, Wilkes GJ, Scanlan SL, Parsonage W, Hickman PE.Use of observed within-person variation of cardiac troponin in emergency department patients for determination of biological variation and percentage and absolute reference change values. *Clin Chem.* 2014; 60: p. 848-54.

15. Ai J, Zhang R, Li Y, Pu J, Lu Y, Jiao J, Li K, Yu B, Li Z, Wang R, Wang L, Li Q, Wang N, Shan H, Li Z, Yang B. Circulating microRNA-1 as a potential novel biomarker for acute myocardial infarction. *Biochem Biophys Res Commun.* 2010; 391: p. 73-7.

16. Kuwabara Y, Ono K, Horie T, Nishi H, Nagao K, Kinoshita M, Watanabe S, Baba O, Kojima Y, Shizuta S, Imai M, Tamura T, Kita T, Kimura T. Increased microRNA-1 and microRNA-133a levels in serum of patients with cardiovascular disease indicate myocardial damage. *Circ Cardiovasc Genet*. 2011; 4: p. 446-54.

17. Care A, Catalucci D, Felicetti F, Bonci D, Addario A, Gallo P, Bang ML, Segnalini P, Gu Y, Dalton ND, Elia L, Latronico MV, Høydal M, Autore C, Russo MA, Dorn GW 2nd, Ellingsen O, Ruiz-Lozano P, Peterson KL, Croce CM, Peschle C, Condorelli G. MicroRNA-133 controls cardiac hypertrophy. *Nat Med.* 2007; 13: p. 613-8.

18. van Rooij E, Sutherland LB, Liu N, Williams AH, McAnally J, Gerard RD, Richardson JA, Olson EN. A signature pattern of stress-responsive microRNAs that can evoke cardiac hypertrophy and heart failure. *Proc Natl Acad Sci U S A.* 2006; 103: p. 18255-60.

19. Corsten MF, Dennert R, Jochems S, Kuznetsova T, Devaux Y, Hofstra L, Wagner DR, Staessen JA, Heymans S, Schroen B. Circulating MicroRNA-208b and MicroRNA-499 reflect myocardial damage in cardiovascular disease. *Circ Cardiovasc Genet*. 2010; 3: p. 499-506.

20. van Rooij E, Sutherland LB, Qi X, Richardson JA, Hill J, Olson EN. Control of stress-dependent cardiac growth and gene expression by a microRNA. *Science*. 2007;316: p. 575-9.

21. Han M, Toli J, Abdellatif M. MicroRNAs in the cardiovascular system. *Curr Opin Cardiol.* 2011;26: p. 181-9.

22. Long G, Wang F, Duan Q, Yang S, Chen F, Gong W, Yang X, Wang Y, Chen C, Wang DW. Circulating miR-30a, miR-195 and let-7b associated with acute myocardial infarction. *PLoS One*. 2012;7: p. e50926.

23. Zhao CH, Cheng GC, He RL, Hong Y, Wan QL, Wang ZZ, Pan ZY. Analysis and clinical significance of microRNA-499 expression levels in serum of patients with acute myocardial infarction. *Genet Mol Res.* 2015; 14: p. 4027-34.

24. Tijsen AJ, Creemers EE, Moerland PD, de Windt LJ, van der Wal AC, Kok WE, Pinto YM. MiR423-5p as a circulating biomarker for heart failure. *Circ Res.* 2010; 106: p. 1035-9.

25. van Rooij E, Sutherland LB, Thatcher JE, DiMaio JM, Naseem RH, Marshall WS, Hill JA, Olson EN. Dysregulation of microRNAs after myocardial infarction reveals a role of miR-29 in cardiac fibrosis. *Proc Natl Acad Sci U S A.* 2008;105: p. 13027-32.

26. Zile MR, Mehurg SM, Arroyo JE, Stroud RE, DeSantis SM, Spinale FG. Relationship between the temporal profile of plasma microRNA and left ventricular remodeling in patients after myocardial infarction. *Circ Cardiovasc Genet*. 2011; 4: p. 614-9.

27. Li R, Yan G, Li Q, Sun H, Hu Y, Sun J, Xu B. MicroRNA-145 protects cardiomyocytes against hydrogen peroxide (H(2)O(2))-induced apoptosis through targeting the mitochondria apoptotic pathway. *PLoS One*. 2012; 7: p. e44907.

28. Kim JH, Park SG, Song SY, Kim JK, Sung JH. Reactive oxygen species-responsive miR-210 regulates proliferation and migration of adipose-derived stem cells via PTPN2. *Cell Death Dis.* 2013;4: p. e588.

29. Aurora AB, Mahmoud AI, Luo X, Johnson BA, van Rooij E, Matsuzaki S, Humphries KM, Hill JA, Bassel-Duby R, Sadek HA, Olson EN. MicroRNA-214 protects the mouse heart from ischemic injury by controlling Ca(2)(+) overload and cell death. *J Clin Invest.* 2012;122: p. 1222-32.

30. Wu C, So J, Davis-Dusenbery BN, Qi HH, Bloch DB, Shi Y, Lagna G, Hata A. Hypoxia potentiates microRNA-mediated gene silencing through posttranslational modification of Argonaute2. *Mol Cell Biol.* 2011;31: p. 4760-74.

31. Wang K, Li PF. Foxo3a regulates apoptosis by negatively targeting miR-21. *J Biol Chem.* 2010; 285: p. 16958-66.

32. Vacchi-Suzzi C, Bauer Y, Berridge BR, Bongiovanni S, Gerrish K, Hamadeh HK, Letzkus M, Lyon J, Moggs J, Paules RS, Pognan F, Staedtler F, Vidgeon-Hart MP, Grenet O, Couttet P. Perturbation of microRNAs in rat heart during chronic doxorubicin treatment. *PLoS One*. 2012;7: p. e40395.

33. Horie T, Ono K, Nishi H, Nagao K, Kinoshita M, Watanabe S, Kuwabara Y, Nakashima Y, Takanabe-Mori R, Nishi E, Hasegawa K, Kita T, Kimura T. Acute doxorubicin cardiotoxicity is associated with miR-146a-induced inhibition of the neuregulin-ErbB pathway. *Cardiovasc Res.* 2010;87: p. 656-64.

34. Fu J, Peng C, Wang W, Jin H, Tang Q, Wei X. Let-7 g is involved in doxorubicin induced myocardial injury. *Environ Toxicol Pharmacol.* 2012;33: p. 312-7.

35. Andersen CL, Jensen JL, Ørntoft TF. Normalization of real-time quantitative reverse transcription-PCR data: a model-based variance estimation approach to identify genes suited for normalization, applied to bladder and colon cancer data sets. *Cancer Res.* 2004; 64: p. 5245-50.
